# Does dual‐tasking affect the ability to generate anticipatory postural adjustments in young adults?

**DOI:** 10.1002/ejsc.12083

**Published:** 2024-03-18

**Authors:** Angeliki Vazaka, Zoe Franklin, Richard Mills

**Affiliations:** ^1^ Department of Sport and Exercise Sciences Faculty of Science & Engineering Manchester Metropolitan University Manchester UK; ^2^ Manchester Metropolitan University Institute of Sport Manchester UK

**Keywords:** balance mechanisms, dual tasking, postural control

## Abstract

The aim of this study was to investigate how additional cognitive tasks (Stroop test and counting backwards task) influence young adults' ability to generate appropriate postural responses while standing on a continuously oscillating platform. Twenty young adults (25.95 ± 2.97 years) stood on a moving platform which was translated in the anterior–posterior direction at three different frequencies (0.10, 0.25 and 0.50 Hz) in three dual‐task conditions (counting backwards task, a Stroop task or no additional cognitive task). Postural muscle onset latencies and tonic activity levels of the leg muscles were measured through surface electromyography; the number of steps taken and cognitive errors made were recorded. Results showed no significant differences in muscle activity between dual and single‐tasking conditions nor between the two dual tasking conditions. Cognitive errors were made in the counting backwards task but not the Stroop task. A frequency effect was identified with participants showing greater tonic activity in rectus femoris (*p* = 0.012), gastrocnemius medialis (*p* = 0.016) and bicep femoris (*p* = 0.043) at 0.5 Hz, as well as earlier muscle activation in tibialis anterior, gastrocnemius medialis and bicep femoris (*p* < 0.001) at 0.50 Hz. Transition and steady state muscle onset latencies were only significantly different for gastrocnemius medialis at 0.25 Hz (*p* = 0.001). Dual tasking did not seem to influence anticipatory postural adjustments in young adults; however, perturbation intensities did. The differences observed in the number of cognitive errors made could be indicative of the regional cortical activations and overlapping demand for resources interfering with balance control, though cortical activation was not recorded. Future research should include detailed cognitive behavior, including cortical activations and task reaction times to better understand the allocation of attentional resources during perturbed balance dual tasking.

## Highlights

- Directionally specific muscle onset latencies in the tibialis anterior, gastrocnemius medialis and bicep femoris were triggered sooner at 0.50 Hz than in the other lower frequencies.
- Postural responses were largely unchanged between single and dual tasking, suggesting that for young adults, dual‐tasking appears to have no more strength to alter postural control responses than altered support surface stability alone.
- Only the counting backwards dual‐task condition elicited cognitive errors, suggesting a potential overlapping demand for resource allocation between tasks, though this will need to be confirmed in future work with observation of cortical activation (e.g., through EEG or fNIRS)

## 1 INTRODUCTION

Balance is commonly deﬁned as the ability to keep the body's center of gravity within its base of support and can be characterized as either static or dynamic balance (Goldie et al., 1989). Humans are regularly faced with externally induced perturbations that challenge balance (e.g., standing on a moving bus). On such occasions, postural muscles must be activated in order to restore the center of mass (CoM) stability. Typically, three strategies are used: two feet‐in‐place strategies—the ankle and the hip strategies (for small to medium sized perturbations)—and a stepping strategy (for large perturbations), which can be used either separately or combined to restore balance (Nashner et al., 1985). The speed of the perturbation also affects the choice of the response used (Hwang et al., 2009).

When faced with discrete unexpected perturbations, such as a single movement of a support surface resembling a trip or slip, compensatory postural adjustments (i.e., activation of postural muscles *after* the perturbation has occurred) are used to correct for the shift in CoM (Welch et al., 2008). However, when a perturbation is predictable, anticipatory postural adjustments are made by activating postural muscles *in advance of* the upcoming disturbance, therefore reducing the need for large compensatory postural adjustments after the perturbation (Frank et al., 1990; Pavol et al., 2002). Anticipatory postural adjustments can also be elicited during whole‐body movements, such as the initiation of gait (Crenna et al., 1991; Honeine et al., 2016). The oscillating platform paradigm, where the support surface is perturbed at various frequencies and amplitudes, provides insight into the ability to switch between anticipatory and compensatory mechanisms. A compensatory response is stimulated by the initial perturbation, and as the platform continues to oscillate, a switch to an anticipatory mechanism is observed (Schmid et al., 2011). For example, Bugnariu & Sveistrup (2006) showed that younger adults exhibit a shift to earlier postural muscle onset latencies (as obtained by surface electromyography, EMG) within three to five cycles of externally induced sinusoidal platform oscillation indicative of anticipatory responses, while older adults continued to exhibit slower onset latencies indicative of compensatory responses. Further, Mills and Sveistrup (2018) found that these postural responses (i.e., postural muscle onset latencies) in children and adolescents are similar to those used by older adults. Prior experience of the perturbation has also been shown to affect anticipatory postural adjustments (Van Ooteghem et al., 2009), resulting in decreased center of pressure (CoP) displacement and earlier postural muscle activation (Kennedy et al., 2013).

Circumstances requiring the processing of motor and cognitive tasks simultaneously (i.e., dual tasking) constitute a significant component of the modern busy lifestyle (e.g., talking on the phone while standing or walking). During dual tasking, the performance of one or both tasks can decrease if the task requirements exceed the available cognitive capacity. Postural and cognitive tasks compete for attentional resources; balance performance is compromised when cognitive and motor tasks are performed simultaneously (Andersson GY et al., 1998; Pellecchia, 2003). Rankin et al. (2000) tested the effects of a cognitive task (counting backwards by 3 s) on the neuromuscular response characteristics of reactive balance control in both young and older adults. Using EMGs on the core and lower limbs, the authors observed that for both older and young adults, onset latency of postural muscle responses did not change during dual tasking; however, the amplitude of postural muscle activity was affected by the counting backwards task with older adults showing greater reduction than young adults. The decline of muscle activity when the counting backwards task was performed suggests that less attentional capacity was available for balance control during dual tasking. Reduced balance performance during dual tasks is mostly observed in older populations (e.g., larger sway areas) but has also been observed in young adults (Riley et al., 2003; Swan et al., 2004), however with mixed results. For example, Beretta et al. (2019) observed that young adults reduced their CoP sway during dual tasking on a moving platform. Their cognitive task was to report how many times a preset number appeared in the audio. On the other hand, Dault et al. (2001) found that young adults improved postural stability as seen by their increased frequency and decreased amplitude of sway when three modified versions of the Stroop task (of different difficulty levels) were added.

The types of cognitive task performed during dual tasking can influence balance performance. Maylor et al. (2001) found that compared to the spatial Brooks' task (participants were instructed to place consecutive numbers in a 4 × 4 grid, e.g. `In the next square to the right/left/up/down put a 2′), both young and older adults had higher sway velocity when performing the non‐spatial Brooks' task during quiet stance. The non‐spatial task was based on the stimuli from the spatial task replacing the words right, left, up and down with the words quick, slow, good and bad, respectively. During quiet stance, a counting backwards mental task also induced postural sway in older adults, while the Stroop test did not (Jamet et al., 2004). For the correct execution of visuo‐verbal tasks, such as the Stroop test, accurate visual fixation and focussed attention on the colored word are necessary (MacLeod, 1991). Therefore, it is possible that a stable visual landmark usage can compensate for the adverse effects of added cognitive load on balance. Conversely, counting backwards does not require gaze fixation for its execution and instead may cause a person to mentally imagine the arithmetic task rather than focusing on a stable visual landmark, destabilizing postural control (Jamet et al., 2004). Dual tasking can also affect the balance strategy used. For example, the use of the stepping strategy is elicited following discrete perturbations combined with a secondary cognitive task (i.e. counting backwards by 3) (Rankin et al., 2000).

To date, little is known about the impact of cognitive tasks on perturbed balance and whether different types of cognitive tasks elicit different balance mechanisms. Therefore, the aim of the current study was to investigate how dual tasking influences young adults' ability to generate anticipatory postural adjustments while standing on a continuously oscillating platform. It was hypothesized that participants would (i) display delayed postural muscle onset latencies as well as greater tonic activity, make more cognitive errors and take more steps during dual tasking conditions compared to the single task condition; (ii) not be able to shift from reactive to anticipatory postural mechanisms (as evidenced by the timing of the activations) during dual tasking when compared to single tasking and (iii) display different muscle onset latencies and tonic activity between two cognitive task conditions.

## 2 METHODS

Twenty young adults (11 females and 9 males, recruited from the university student population) participated in this study. Mean (±SD) age was 25.95 (±2.97) years, height was 172.69 (±8.75) cm and mass was 70 (±14.10) kg. All participants were free from neuromuscular disorder and had no existing or unresolved injuries that could limit movement. The study was reviewed and approved by the institutional ethics committee. Written consent was obtained from each participant before taking part in the experiment.

### 2.1 Procedure

Participants attended the laboratory at Manchester Metropolitan University for one 90‐min session. They were asked to stand eyes open, bare feet shoulder‐width apart on a custom‐built moveable platform (80 × 60 cm), driven by an electromagnetic actuator and controlled through Copley Controls CME2 software (Canton, USA). The platform was translated at 20 cm peak‐to‐peak in the anterior–posterior direction at three different frequencies (0.10, 0.25 and 0.50 Hz) all from stationary start. Trials for each frequency were 100 s long and consisted of at least 10 cycles at 0.10 Hz, 20 cycles at 0.25 Hz and 40 cycles at 0.50 Hz.

**FIGURE 1:**
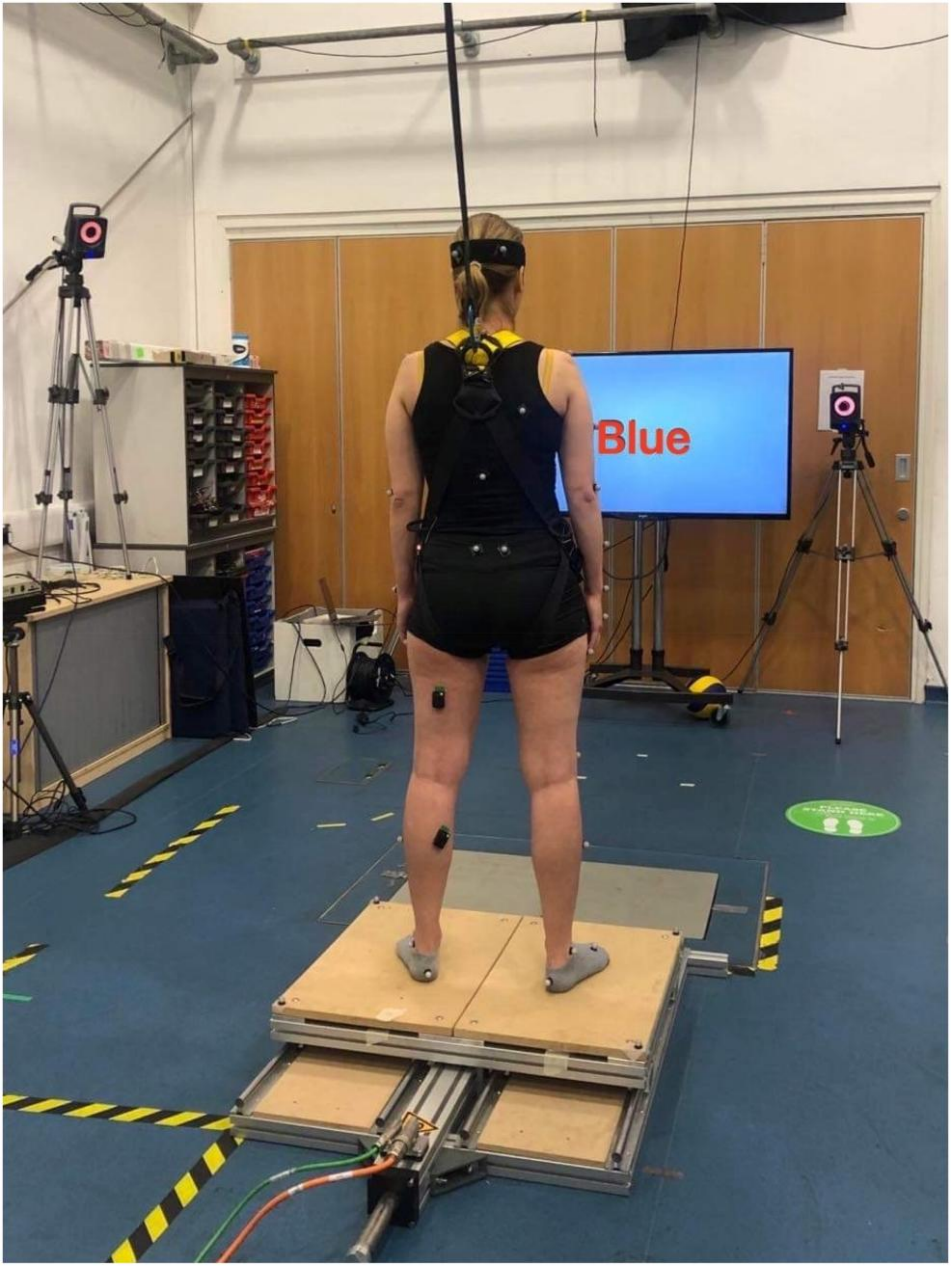
Experimental setup. Participant performing the Stroop Task (the word ‘blue’ in red text) while stood on the moving platform.

Participants were tested under three conditions: (i) Stroop Test (ST), (ii) counting backwards (CB) and (iii) no cognitive task (NCT). For the ST, participants were presented with colored words, representing color names that were different from the printed colors. They were instructed to name the colors of the text as quickly as possible. For example, if the word was “yellow” and was printed in red ink, the correct answer would be “red”. The ST was performed using PsychoPy software (Peirce et al., 2019). Words appeared on the screen one at a time every 4 seconds. For the CB condition, participants were given a random number over 100, from which they counted backwards by seven (Maclean et al., 2017) as fast and as accurately as possible for the duration of the trial. Numbers over 100 were chosen to ensure participants would not count below zero. An audio recording device was used to record their answers so that the number of errors for both cognitive tasks could be counted. For the NCT and CBT conditions, participants were instructed to focus on a cross projected on a screen in front of them at the same level and distance as the ST visuals. In all conditions, if a step was taken, participants were instructed to regain their balance and return to their initial position. The number of steps taken by each subject at each frequency was documented. Participants also performed the CB and ST, while stood in quiet stance (30 s) to compare dual tasking cognitive performance (i.e., number of errors made) to single tasking. Two trials of each condition in randomized order were performed. Participants were given 1 minute rest between trials and were afforded additional opportunities to rest if they felt necessary. Participants were also equipped with a harness attached to an auto belay from the ceiling in case the perturbation caused a fall. Postural muscle activation was recorded via surface electromyography (EMG) (Delsys Trigno, Delsys Inc, USA) (1000 Hz). Surface electrodes were placed on the skin over the muscle belly of the tibialis anterior (TA), gastrocnemius medialis (GM), rectus femoris (RF) and bicep femoris (BF) on the left side of the body following SENIAM guidelines. Figure 1 depicts the experimental setup.

### 2.2 Data analysis

**FIGURE 2:**
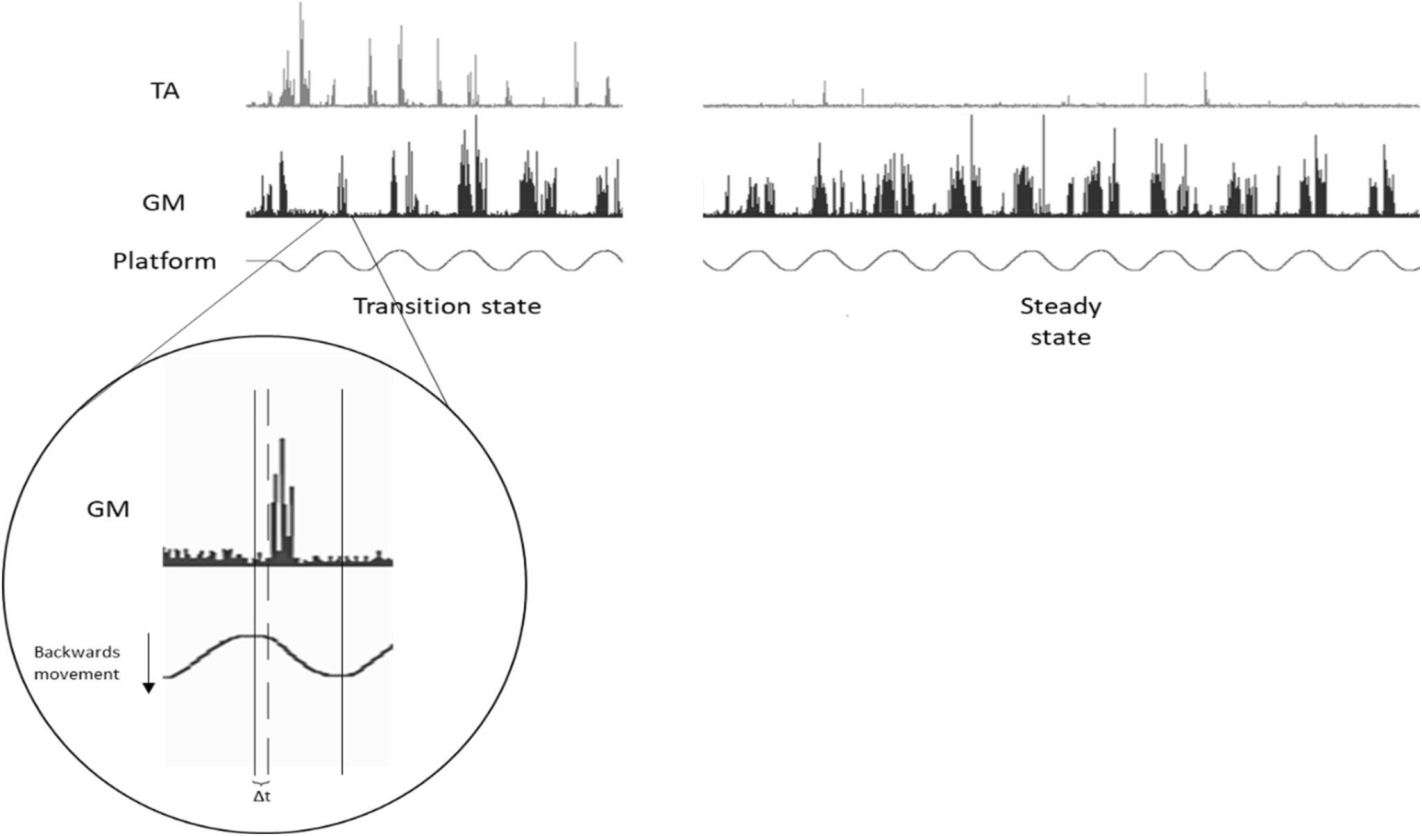
Perturbation protocol depicting platform oscillation at 0.25 Hz with corresponding EMG signals from tibialis anterior (TA) and gastrocnemius medialis (GM) during transition and steady states. The enlarged EMG signal of the GM is an example of one backwards platform movement (1/2 cycle), indicated by the two black vertical lines: the first black line indicates the start of the backwards movement and the second indicates the end of the backwards movement and the beginning of the forward movement. The dashed line indicates the start of the muscle activation and the time between the first black line and the dashed line (Δt) is the muscle onset latency.

Data were processed offline. In each trial, the first 3–5 consecutive cycles without stepping at each frequency were considered “transition state” periods, where reactive postural responses were expected. In the last half of the trial, a series of 3–5 consecutive cycles without stepping at 0.10 Hz and 8–10 consecutive cycles without stepping for the remaining frequencies were considered the “steady state” period where anticipatory postural adjustments were likely to occur (Figure 2) (Bugnariu et al., 2006; Mills et al., 2018).

EMG processing was performed in BioProc for Windows software (BioProc for Windows, 2008). Bias in the EMG signals was removed where appropriate and signals fullwave rectified. Postural muscle onset latencies were determined from raw EMG signals. The first burst of activity associated with a perturbation lasting more than 50 ms and greater than two standard deviations above the within‐trial baseline (i.e., quiet period with no activity) indicated muscle onset latency activity (Mills et al., 2018). Baseline was determined for each trial during the quiet stance period before platform movement initiation. To be included in the calculations of group muscle activity, responses had to be present in at least 30% of the directionally specific perturbations at each frequency (i.e., anterior muscles for backward‐to‐forward changes of direction and posterior muscles for forward‐to‐backward changes of direction) for transition state periods and 50% for steady state periods. For the 0.10 Hz frequency, the recruitment threshold was reduced to 20% of perturbations for both transition and steady state. Considering that each cycle's duration was frequency dependent, muscle onset latencies were determined and expressed as a percentage of half‐cycle time (i.e., platform movement from one extreme position to the other). If muscle activity began after zero, latencies were coded as positive indicating reactive responses. If muscle activity began before zero, latencies were coded as negative indicating anticipatory responses (Bugnariu et al., 2006).

Tonic postural muscle activity was determined as follows: in each trial, a period of inactivity (i.e., no muscle bursts occurring) was identified in transition and steady state. For each participant, this was then compared to their “baseline” which was determined during a period in steady state in NCT at 0.10 Hz where no burst activity was present. Tonic activity for each trial was then expressed as a percentage of the baseline tonic activity level in NCT steady state at 0.10 Hz.

### 2.3 Statistical analysis

Descriptive analysis was used to summarize the participant demographics and stepping data. As two trials were performed for each condition, outcome measures were averaged across these two trials where applicable (e.g., step counts and cognitive errors were not averaged, but rather presented as total counts). IBM SPSS Statistics version 26 was used to analyze onset latencies and tonic activity levels. The onset latency data were determined to be parametric in all muscles (Shapiro–Wilk and Levene's tests) apart from the TA, while tonic activity was determined to be non‐parametric for all muscles. To determine main effects and interactions between the different frequencies, conditions and states, a 3 (frequencies) × 3 (conditions) × 2 (transition and steady state period) factorial ANOVA was used for the parametric data, while Bonferroni post hoc tests were performed to identify significant differences in pairwise comparisons. Accepted level of significance was set at *p* < 0.05. For the tonic activity and the TA onset latencies that were determined as non‐parametric, Wilcoxon sign ranked tests were run for the pairwise comparisons where main effects or interactions were identified in the ANOVA. Cognitive data were analyzed using a Friedman test to compare the cognitive errors between frequencies and conditions, while Wilcoxon sign ranked tests were run for the pairwise comparisons where main effects or interactions were observed. To correct for multiple tests, *α* was adjusted by dividing 0.05 by the number of comparisons.

## 3 RESULTS

### 3.1 Onset latencies

For the tibialis anterior, significant frequency effects were found (*F* (2, 95) = 17.134, *p* < 0.001, *η*_*p*_^2^ = 0.265) and a Wilcoxon signed rank test revealed significant difference between 0.25 and 0.50 Hz (*Z* = −4.220, *p* < 0.001, mean score = 11.36) with onset latencies occurring earlier at 0.50 Hz (*M* = −2.700, SD = 8.1% half cycle). Significant interaction effects between frequency and state were also found (*F* (2, 95) = 3.514, *p* = 0.034, *η*_*p*_^2^ = 0.069). A Wilcoxon signed rank test showed significant differences in transition state between 0.25 and 0.50 Hz (*Z* = −3.645, *p* < 0.001, mean score = 10.93) with onset latencies occurring earlier at 0.50 Hz (*M* = 4.80, SD = 1% half cycle) compared to 0.25 Hz (*M* = 14, SD = 2.2% half cycle). No main effects were found for the Quadriceps.

Significant frequency effects were found for the gastrocnemius medialis (*F* (2, 223) = 68.410, *p* < 0.001, *η*_*p*_^2^ = 0.38) with post hoc comparisons identifying significant differences between 0.10 and 0.25 Hz (*p* < 0.001) with onset latencies occurring earlier at 0.10 Hz (*M* = −4.46, SD = 1% half cycle) compared to 0.25 Hz (*M* = 9.18, SD = 2.5% half cycle). Significant differences were also observed between 0.25 and 0.50 Hz (*p* < 0.001) with muscle onset latencies occurring earlier at 0.50 Hz (*M* = −9.54, SD = 3.3% half cycle) and between 0.10 and 0.5 Hz (*p* = 0.003) with muscle onset latencies occurring earlier at 0.50 Hz. A significant interaction effect between frequency and state was also found for the gastrocnemius medialis (*F* (2, 223) = 4.244, *p* = 0.016, *η*_*p*_^2^ = 0.037), and post hoc comparisons revealed that transition state was significantly different to steady state at 0.25 Hz (*p* = 0.001) with onset latencies occurring earlier in steady state (*M* = 5.17, SD = 1.6% half cycle) than in transition state (*M* = 13.19, SD = 2.2% half cycle).

Significant frequency effects were also seen in the bicep femoris (*F* (2, 100) = 8.944, *p* < 0.001, *η*_*p*_^2^ = 0.152). Post hoc comparisons identified significant differences between 0.10 and 0.25 Hz (*p* < 0.001) with onset latencies occurring earlier at 0.10 Hz (*M* = −2.90, SD = 1.8% half cycle) compared to 0.25 Hz (*M* = 9.5, SD = 4.6% half cycle). Significant differences were also seen between 0.25 and 0.50 Hz (*p* < 0.001) with onset latencies occurring earlier at 0.50 Hz (*M* = −1.34, SD = 3.9% half cycle).

**FIGURE 3:**
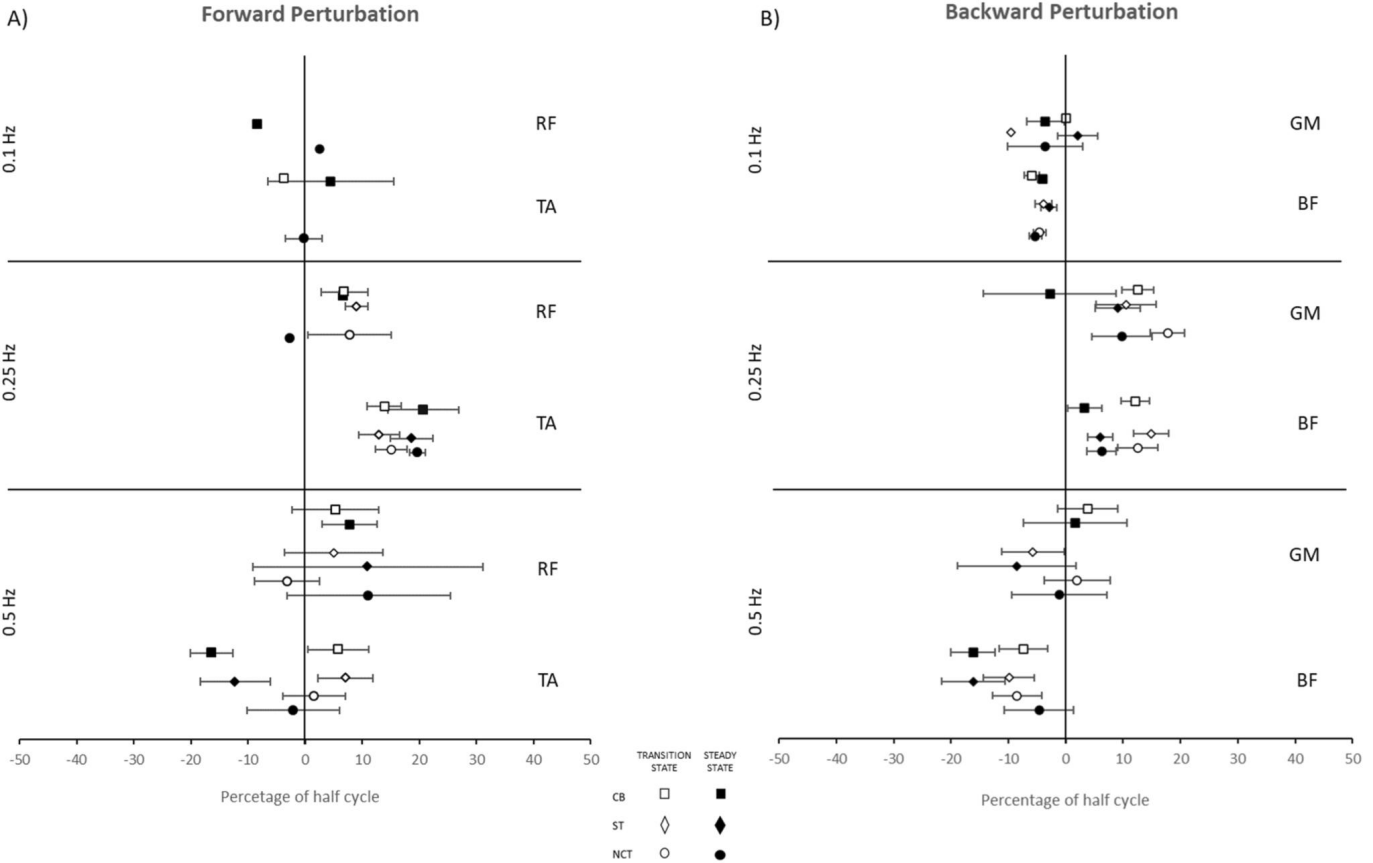
Postural muscles onset latencies (mean ± SE) during forward (A) and backward (B) perturbations at the three frequencies of platform oscillation. Onset latencies are expressed as a percentage of half cycle time for muscles normally associated with forward (TA and RF in panel A) or backward (GM and BF in panel B) perturbations. Results from transition and steady states are represented by open and filled icons, respectively, while counting backwards (CB), Stroop test (ST) and no cognitive task (NCT) conditions are represented by squares, diamonds and circles, respectively. Zero (0) represents the time at which the platform changed direction; the platform begins to slow down at the 50% half cycle mark. Where latencies begin after zero (0), reactive responses are indicated by positive values. Where muscle activity begins before zero, latencies are negative, indicating anticipatory responses. Transition and steady state icons are offset for clear visual presentation.

No main effects were identified between dual and single tasking for any muscle. Figure 3 illustrates the onset latencies of each muscle at all three frequencies and conditions as well as transition and steady state. Figures depicting muscle onset latencies comparing transition and steady states can be found in the online supplementary material.

### 3.2 Tonic activity

Tonic postural muscle activity was expressed as a percentage of the baseline tonic activity level in NCT steady state at 0.10 Hz. No main effects were found for tonic activity in the tibialis anterior, while significant frequency effects were found for tonic activity in the Rectus Femoris (*F* (2, 339) = 4.474, *p* = 0.012, *η*_*p*_^2^ = 0.026). Wilcoxon signed rank tests revealed significant differences between 0.1 and 0.50 Hz (*Z* = −3.862, *p* < 0.001, mean score = 62.11) with tonic activity being greater at 0.50 Hz (*M* = 177% baseline, SD = 79.2), as well as between 0.25 and 0.50 Hz (*Z* = −3.468, *p* = 0.001, mean score = 61.21) where tonic activity was again greater at 0.50 Hz compared to 0.25 Hz (*M* = 144.1% baseline, SD = 29.5).

Significant frequency effects were also found for tonic activity in the gastrocnemius medialis (*F* (2, 338) = 4.168, *p* = 0.016, *η*_*p*_^2^ = 0.024). Wilcoxon signed rank tests showed significant differences between 0.25 and 0.50 Hz (*Z* = −2.776, *p* = 0.006, mean score = 61.98) with tonic activity being greater at 0.50 Hz (*M* = 274.8% baseline, SD = 201.4) compared to 0.25 Hz (*M* = 184.4% baseline, SD = 77.5).

For the tonic activity in the bicep femoris, significant interaction effects between frequency and condition were found (*F* (4, 339) = 2.488, *p* = 0.043, *η*_*p*_^2^ = 0.029). Wilcoxon signed rank tests identified significant differences in NCT between 0.25 and 0.50 Hz (*Z* = −2.466, *p* = 0.014, mean score = 20.79) with tonic activity being greater at 0.50 Hz (*M* = 291% baseline, SD = 3.5) compared to 0.25 Hz (*M* = 127% baseline, SD = 21.9). No main effects were identified between dual and single tasking for any muscle. Figures depicting tonic activity can be found in the online supplementary material.

### 3.3 Cognitive errors

The total number of cognitive errors, the number of participants who made errors and the range of errors made during the trial duration for each frequency and condition are presented in Table 1. No errors were made in the ST, regardless of frequency, while in CB, the fewest errors were made during quiet stance. A Friedman test revealed significant interaction effect between cognitive tasks and frequencies (*χ* ^2^ (7) = 101.167, *p* < 0.001). Post hoc analysis with Wilcoxon signed‐rank tests was conducted and showed significant differences between CB and ST at 0.10 Hz (*Z* = −3.633, *p* < 0.001, mean score = 0) at 0.25 Hz (*Z* = −3.635, *p* < 0.001, mean score = 0) and at 0.50 Hz (*Z* = −3.739, *p* < 0.001, mean score = 0). Significant differences were also found for the CB task between quiet stance and 0.10 Hz (*Z* = −3.184, *p* = 0.001, mean score = 9.57), 0.25 Hz (*Z* = −3.152, *p* = 0.002, mean score = 9.50) as well as 0.50 Hz (*Z* = −3.400, *p* = 0.001, mean score = 9.94). No significant differences were found for the ST between frequencies.

**TABLE 1:** Stepping responses and cognitive errors at each frequency and condition.

			Frequency			
			Quiet stance	0.1 Hz	0.25 Hz	0.5 Hz
Stepping responses	CB	Total no. steps	n/a	‐	6	68
		No. participants	n/a	‐	1	13
		Range	n/a	‐	6	1–10
	ST	Total steps	n/a	‐	8	39
		No. participants	n/a	‐	2	7
		Range	n/a	‐	3–5	2–14
	NCT	Total steps	n/a	‐	5	25
		No. participants	n/a	‐	2	7
		Range	n/a	‐	1–4	1–7
Cognitive errors	CB	Total errors	19	74	72	73
		No. participants	8	17	17	18
		Range	1–3	1–12	1–13	1–9
	ST	Total errors	‐	‐	‐	‐
		No. participants	‐	‐	‐	‐
		Range	‐	‐	‐	‐
	NCT	Total errors	n/a	n/a	n/a	n/a
		No. participants	n/a	n/a	n/a	n/a
		Range	n/a	n/a	n/a	n/a

*Note*: The total number of steps taken (top) and cognitive errors made (bottom), the number of participants who stepped immediately after the initiation of platform oscillation (top) or made cognitive errors (bottom) and ranges at each frequency and condition are presented here. Dashes (‐) indicate no steps taken or errors made.

Abbreviation: n/a, not applicable.

### 3.4 Stepping responses

The total number of steps taken, the number of participants who stepped and the ranges of steps taken at each frequency and each condition are also presented in Table 1. Stepping data were not statistically analyzed and the numbers presented are raw counts. Though not presented in Table 1, some falls were recorded: four participants stepped off the platform when they were introduced to the 0.50 Hz perturbation for the first time. Trials were then stopped and restarted to allow participants to safely step back on to the platform.

## 4 DISCUSSION

The aim of this study was to identify whether dual tasking would affect young adults' ability to generate anticipatory postural adjustments. Our hypotheses were only partially supported (i) postural muscle onset latencies and tonic activity levels did not differ between single and dual‐task conditions, but the more cognitive errors and total steps taken were observed in the dual‐task conditions; (ii) the addition of cognitive tasks did not affect the ability to generate appropriate anticipatory postural adjustments and (iii) while more cognitive errors were made in the CB task, there were no differences observed in myoeletric activity between dual‐task conditions.

### 4.1 High‐frequency perturbations are compensated through earlier muscle activation and increased tonic activity levels

The first hypothesis (that participants would display delayed postural muscle onset latencies, greater tonic activity, take more steps and make more cognitive errors during dual tasking compared to the single tasking condition) was partially supported. Postural muscle onset latencies and tonic activity levels were expected to be different between conditions; however, this was not entirely supported. Higher tonic activity indicates that in the most destabilizing frequency (0.50 Hz), participants were able to control posture by adopting a functional joint stiffening method (Needle et al., 2014). This has previously been hypothesized to be related to the nature and difficulty levels of the tasks (Albertsen et al., 2017; Dault et al., 2001). For instance, when participants performed the Stroop task and adopted a seesaw stance compared to shoulder width stance, mean sway frequency was increased, suggesting stiffness was increased to deal with the additional cognitive demands (Dault et al., 2001). In the current study, the observed increase in tonic activity could be associated with an element of threat observed at the more challenging frequency, similar to the stiffening strategy adopted when surface height is elevated (Adkin et al., 2000; Carpenter et al., 2001). However, this was only evidenced in the NCT condition. It has been reported that postural control is automated when attention is focussed on cognitive tasks, sometimes resulting in improved postural performance (Huxhold et al., 2006; McNevin et al., 2002; Wulf et al., 2001, 2004). Therefore, higher tonic activity of the Hamstrings observed in the NCT at 0.50 Hz could indicate that in the single tasking condition, attention was directed to the postural task, focusing on the “threatening” factor of the high frequency perturbation, leading to a “stiffer” position.

A frequency effect was also observed for onset latencies regardless of conditions. When participants were faced with the lowest frequency, they were able to activate their muscles earlier than at the 0.25 Hz frequency, indicating that the 0.10 Hz frequency was an easy postural task. During platform oscillations at low frequencies, such as 0.10 Hz, postural sway is only slightly increased compared to quiet stance (Sakanaka et al., 2021) and stability can be maintained through the use of visual cues (Dichgans et al., 1976; Lestienne et al., 1977). This would suggest that in the current study, the lower frequency possibly only required minor corrections in CoM sway dealt with by instant muscle activity. Interestingly, muscle onsets at 0.50 Hz occurred earlier than in the lower frequencies. Similar results were found in single‐tasking anticipatory balance (Azzi et al., 2017), whereby large perturbations, caused by suddenly releasing a load attached to the participant's trunk, were compensated for by increasing magnitude and decreasing onset latency of muscle activation compared to lower perturbations. The onset of muscle activation seems to be controlled through anticipatory mechanisms brought about by a state of readiness or “central set” (Jacobs et al., 2007). It might be assumed then, that in the current study, sensory afference signaling a large perturbation triggered pre‐determined postural responses to attend the anticipated requirements of the specific perturbation speed.

As expected, more cognitive errors were also observed in dual tasking when the CB task was performed compared to the baseline errors made in quiet stance. However, the number of errors made between frequencies during dual tasking was not significantly different, possibly indicating that increased postural task difficulty did not have an additional impact on cognitive performance of the CB task. This lack of discrimination between the different frequencies could be attributed to the fact that the reaction time of the CB performance was not measured in this study. It could be the case that at 0.10 Hz, participants performed more, quicker calculations but still had a high number of errors, while at 0.50 Hz, they performed fewer, slower subtractions but with similar number of errors. Since reaction time was not measured, we are unable to conclude that the increased difficulty of the postural task did not have an impact on cognitive performance. Measuring total number of calculations made and the reaction times of answers might have revealed a speed–accuracy trade‐off (Reuss et al., 2015): it is unknown whether participants focussed their attention on doing the task right or doing it fast. Additionally, fewer steps were taken in the NCT since most available attentional resources should be allocated for the recovery and maintenance of balance. As the frequencies increased, the number of steps taken also increased in all conditions, reflecting the increasing difficulty, and thus the increased attentional demands of the postural task. Similar results were observed in a single‐tasking study, where adolescents were exposed to repeated anterior–posterior platform perturbations (Mills et al., 2018).

### 4.2 The influence of perturbation intensity on anticipatory mechanisms

The second hypothesis, that participants would not be able to shift from reactive to anticipatory mechanisms during dual tasking, was not supported. The different conditions did not have an effect, and when all three are combined, transition and steady states were only significantly different for the Gastrocnemius muscle at 0.25 Hz. In the first 5 cycles, participants relied on reactive mechanisms and muscle activation was delayed. As they became accustomed to the perturbation pattern, a shift to anticipatory mechanisms was observed, evidenced by earlier muscle activations in steady state. Muscle activations at 0.10 Hz did not follow any defined trend (e.g., earlier or delayed activations); there appears to be no specific temporal organization, likely because the frequency was not sufficiently threatening to balance and there was no immediate requirement to adapt from transition to steady state. When the speed was threatening to balance at 0.50 Hz, anticipatory mechanisms were triggered at transition state in the hamstrings and gastrocnemius muscles. It has previously been shown that when faced with high frequency platform anterior–posterior translations (0.60 Hz) during single tasking with eyes open, earlier muscle activation occurs within the first or second perturbation cycle and no further adaptations occur (Sozzi et al., 2016). This seems to be the case here, as early muscle activations in the hamstrings and gastrocnemius occurred in the first cycle, indicating the highest frequency required an urgent response from the postural control system to maintain balance.

### 4.3 Dual‐task performance on the oscillating platform may be dependent on task type and resource allocation

The last hypothesis, that onset latencies and tonic activity would be different between dual‐tasking conditions (CB and ST), was made on the basis that the CB task does not require the use of external visual cues for its execution but rather turns one's focus internally negatively impacting postural control, while the ST is a visuo‐verbal task that focuses one's attention externally improving balance (Jamet et al., 2004). However, such differences were not observed. Tonic activity and onset latencies were similar between the two cognitive tasks as well as single tasking. In regard to cognitive performance, the CB task was the only task that yielded errors. Dual task theories suggest that two tasks will interfere with each other if they share common resources (Tombu et al., 2003). Therefore, it could be the case that the cognitive domains involved in the CB task may have overlapped with the cognitive domains involved in perturbed balance. Functional neuroimaging studies have shown that the ST mainly activates the anterior cingulate cortex (Zoccatelli et al., 2010), while the CB task is associated with increased prefrontal cortex activation (Pelicioni et al., 2019). In our study, these neural resources may be devoted toward the balance task leaving fewer resources available to focus on the CB task; hence, the errors were observed. However, we did not record cortical activity in order to identify whether similar brain regions are activated during CB and perturbed balance.

### 4.4 Limitations

The number of trials performed during single and dual tasking conditions might be considered a limitation to this study. It has been found that administering dual task tests, such as the BESS, TGT and CRT, three times and averaging the three trials provided acceptable reliability for clinical use (Manaseer et al., 2020). However, in this study, two trials were performed for each condition and outcome measures were averaged across these two trials where applicable (e.g., step counts were not averaged, but rather presented as total counts). This was determined in pilot testing as it was observed that three trials in each condition would increase data collection time tremendously and would likely have resulted in participants becoming fatigued.

Reaction time of the cognitive task performance during single tasking and dual tasking was not calculated. Cognitive performance was only based on the correct answers of the CB and ST; however, measuring the reaction time of their answers would be a good indicator of attention allocation during the dual tasking conditions. It was observed that as the frequency of the platform perturbation increased, the number of cognitive errors for the CB was similar; however, if the reaction time of their answers had been measured, a speed‐accuracy trade‐off might have been observed. It is unknown whether participants focussed their attention on doing the task right or doing it fast (since the instructions given were ‘count as accurately and as fast as you can’), and it has been found that they can adapt their speed and accuracy between trials and conditions (Reuss et al., 2015); therefore, future studies should take this into account.

In conclusion, our results indicated that dual tasking does not influence the generation of anticipatory postural adjustments in young adults as measured by muscle onset latencies and tonic activity levels, however perturbation intensity does. In the high frequency perturbation, anticipatory mechanisms are generated sooner compared to the lower frequencies as evidenced by earlier muscle onsets latencies, and a stiffer position seems to be adopted as evidenced by increased tonic activity. Regarding the transition and steady states of the perturbations, the lower frequency (0.10 Hz) seemed too easy to require adaptation and the highest frequency (0.50 Hz) was proved large enough to trigger earlier muscle activation from transition state which was then carried to steady state. Since the postural characteristics measured remained unchanged during single and dual tasking, it is assumed that postural control was automated, and the cognitive errors observed in the two tasks reflect their difficulty level. Findings from this study have practical applications and implications for the real world, particularly in training and rehabilitation settings. For example, the potential adaptive strategy to cope with more challenging conditions observed may be relevant for designing interventions aimed at improving postural stability in dynamic environments. These intervention programs could benefit from incorporating perturbation exercises with varying frequencies to enhance effectiveness: in order to maximize challenge to balance, as well as improve anticipatory postural adjustments, higher frequency perturbations could be used. However, before being implemented, future studies should aim to further investigate the balance strategies adopted in various dual‐tasking situations and consider exploring whole body kinetics and kinematics as well as take into account the reaction time of the cognitive task to better understand participants' allocation of attention during perturbed balance dual tasking. Future work should also aim to better understand the effects of different cognitive tasks during perturbed stance on a continuously oscillating platform by including measures of cortical activation levels, such as electroencephalography or functional near‐infrared spectrometry.

## CONFLICT OF INTEREST STATEMENT

No conflict of interest to declare.

## Supporting information

Supporting Information S1
