# Supplementary material for: Does dual‐tasking affect the ability to generate anticipatory postural adjustments in young adults?
Source: Eur J Sport Sci. 2024 Mar 18;24(5):623–33. doi: 10.1002/ejsc.12083 (PMC11235753; doi:10.1002/ejsc.12083)
Supplement: Supplementary file 1 — Supporting Information S1 [file EJSC-24-623-s001.docx]

Appendix 1 – Postural muscle onset latencies for each muscle in transition and steady states.


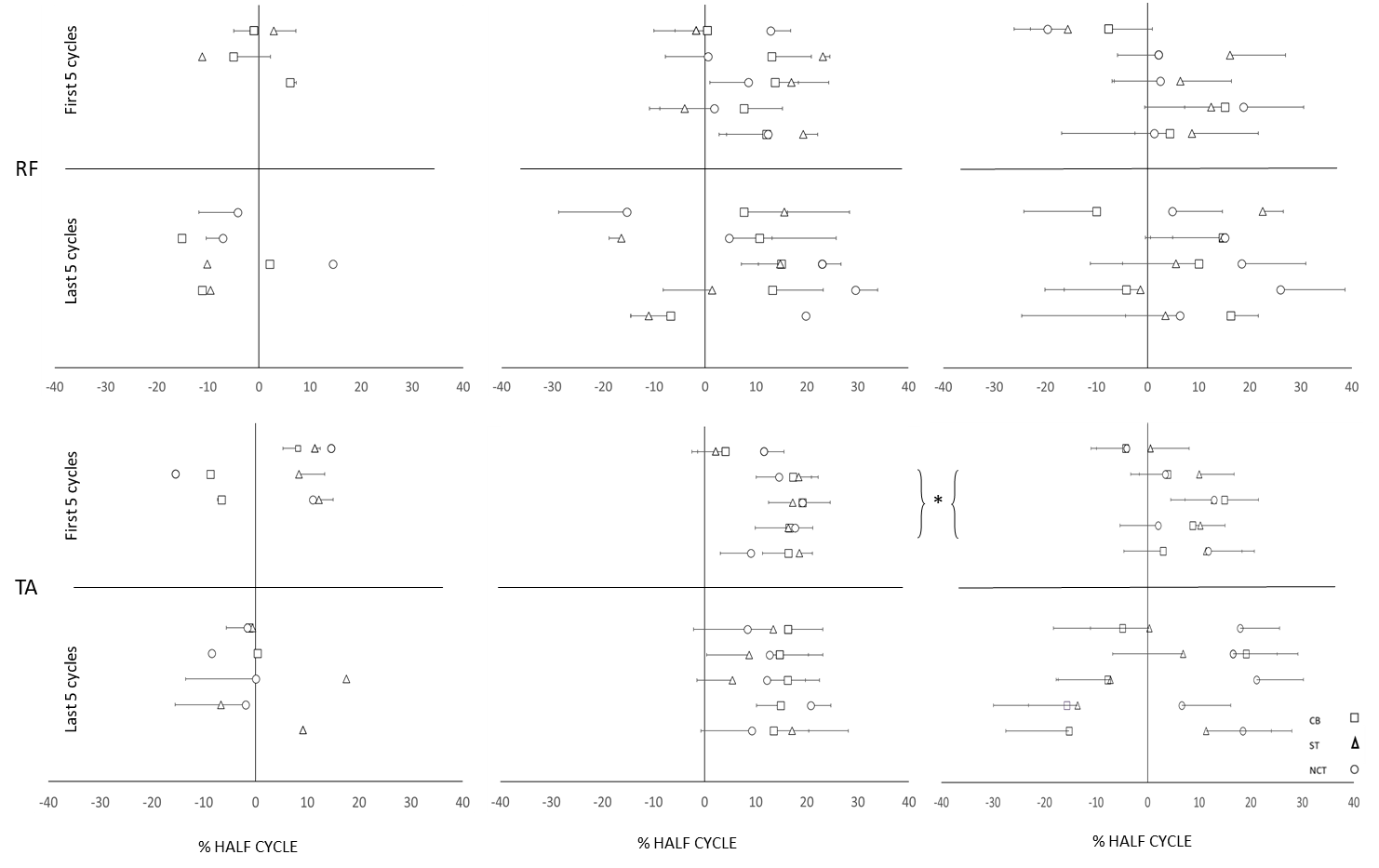


*Supplementary Figure 1A.* Postural muscle onset latencies (mean ± SE) during the first 5 and last 5 cycles for the Rectus Femoris and Tibialis Anterior in each frequency and condition. Onset latencies are expressed as a percentage of half cycle time perturbations. Results from counting backwards (CB), Stroop test (ST) and no cognitive task (NCT) conditions are represented by squares, triangles, and circles, respectively. Zero (0) represents the time at which the platform changed direction; the platform begins to slow down at the 50% half cycle mark. Where latencies begin after zero (0), reactive responses are indicated by positive values. Where muscle activity begins before zero, latencies are negative, indicating anticipatory responses.


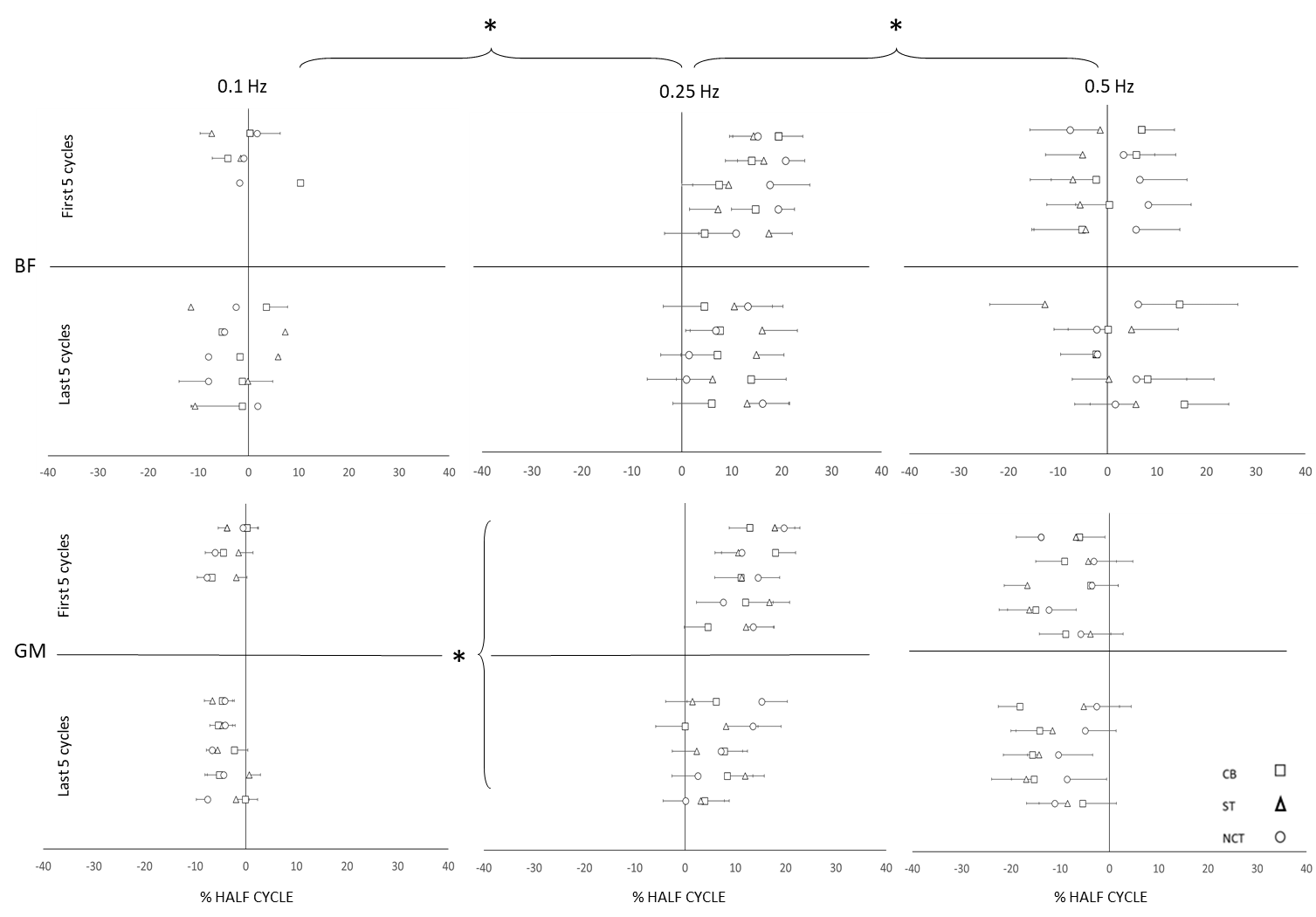


Supplementary Figure 1B. Postural muscle onset latencies (mean ± SE) during the first 5 and last 5 cycles for the Bicep Femoris and Gastrocnemius Medialis in each frequency and condition. Onset latencies are expressed as a percentage of half cycle time perturbations. Results from counting backwards (CB), Stroop test (ST) and no cognitive task (NCT) conditions are represented by squares, triangles, and circles, respectively. Zero (0) represents the time at which the platform changed direction; the platform begins to slow down at the 50% half cycle mark. Where latencies begin after zero (0), reactive responses are indicated by positive values. Where muscle activity begins before zero, latencies are negative, indicating anticipatory responses

**Appendix 2** The percentage of activity bursts per condition by each participant in transition and steady state for each muscle.


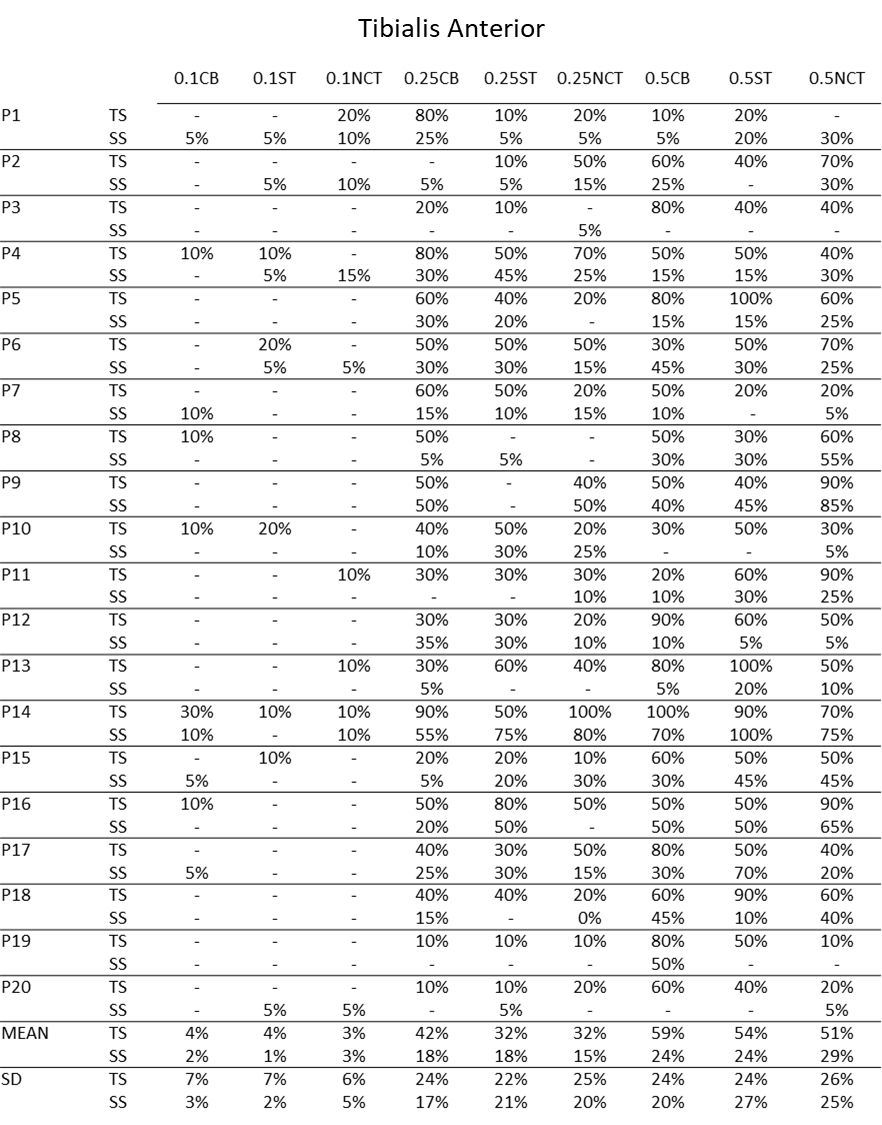


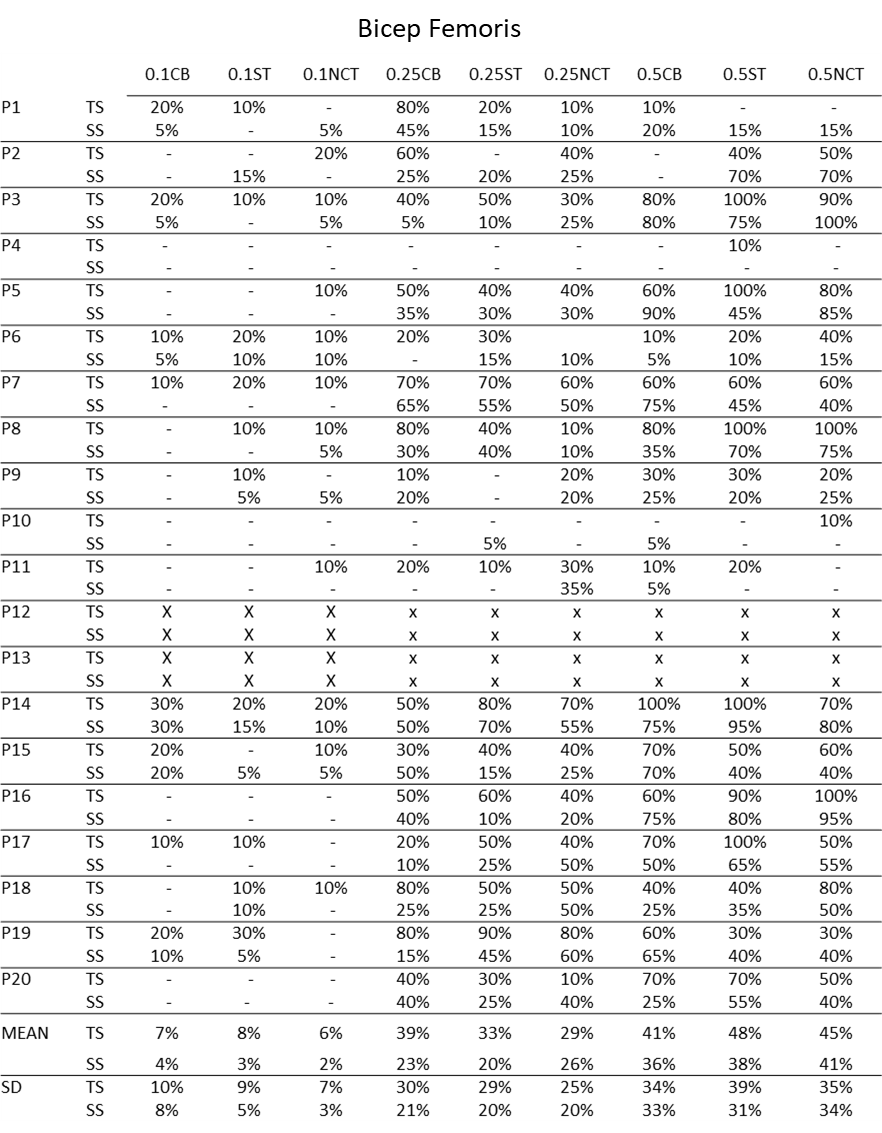


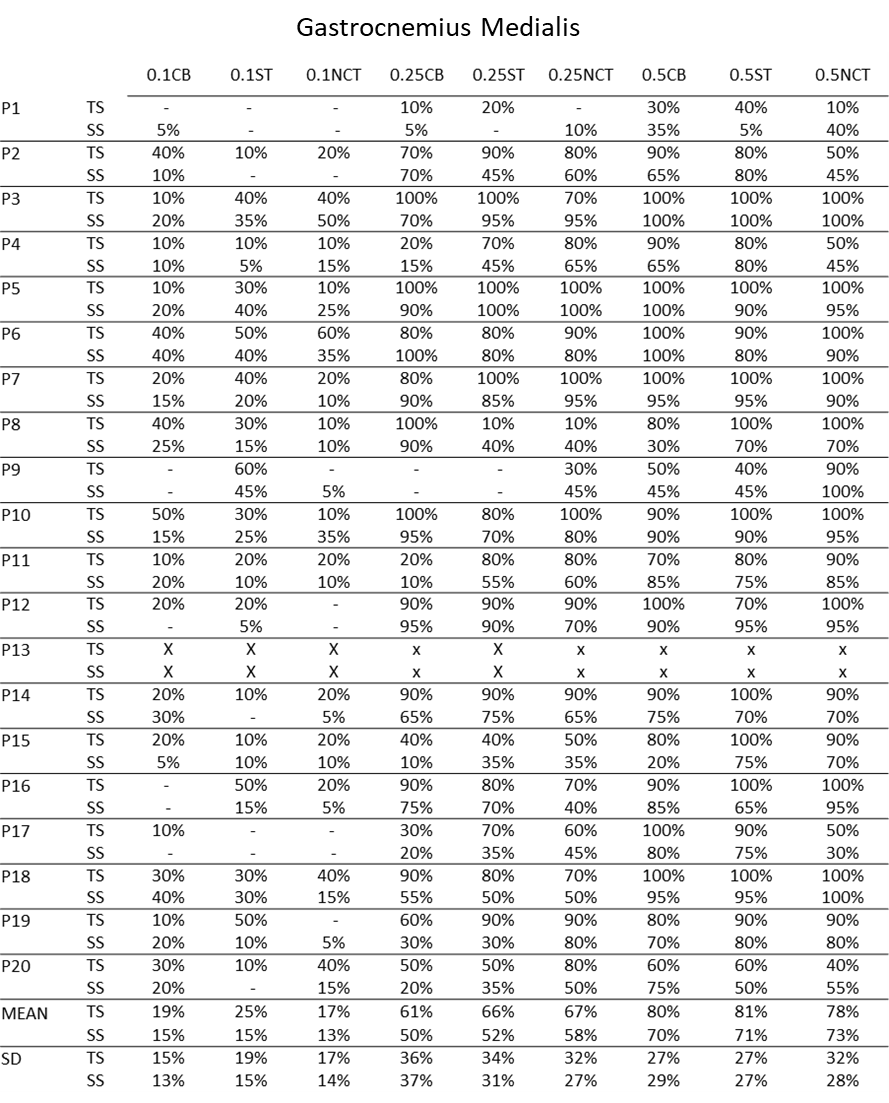


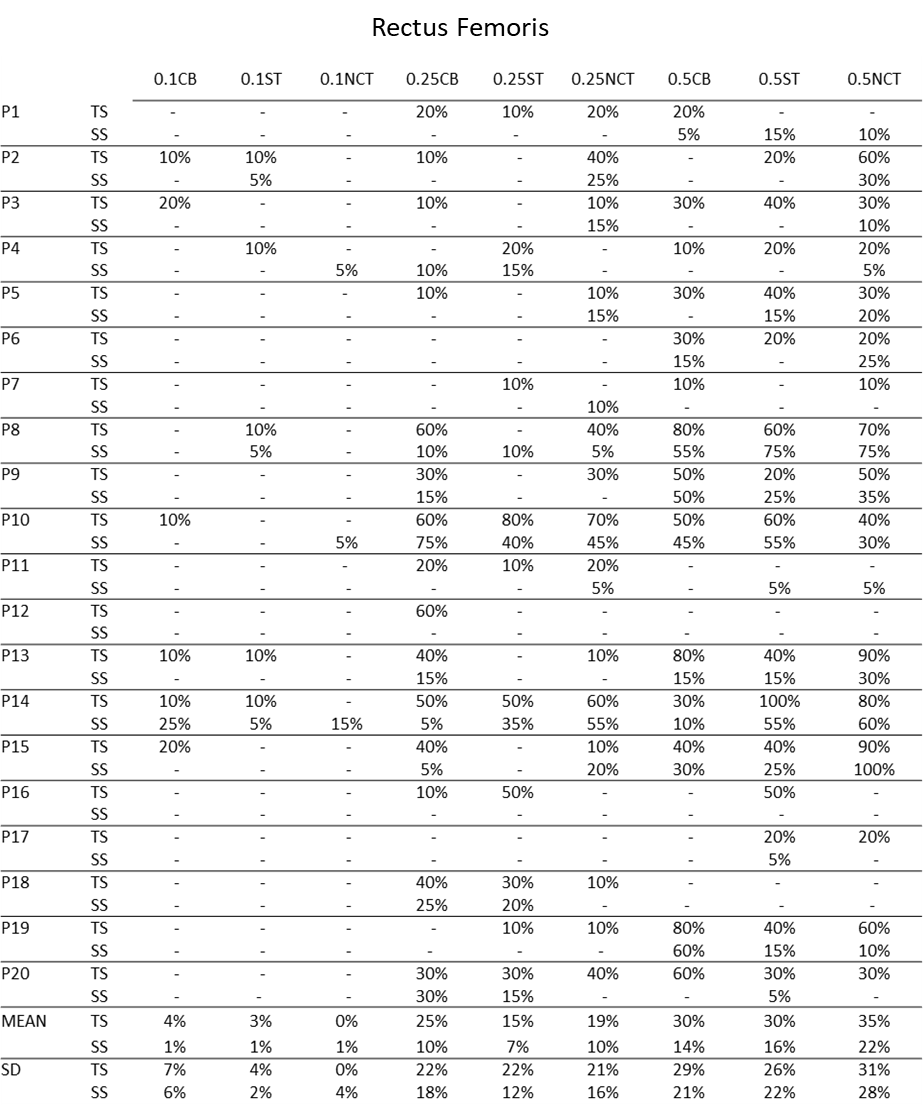


# **Appendix 3** Percent tonic activity in transition and steady state for each muscle (outliers removed for clarity).


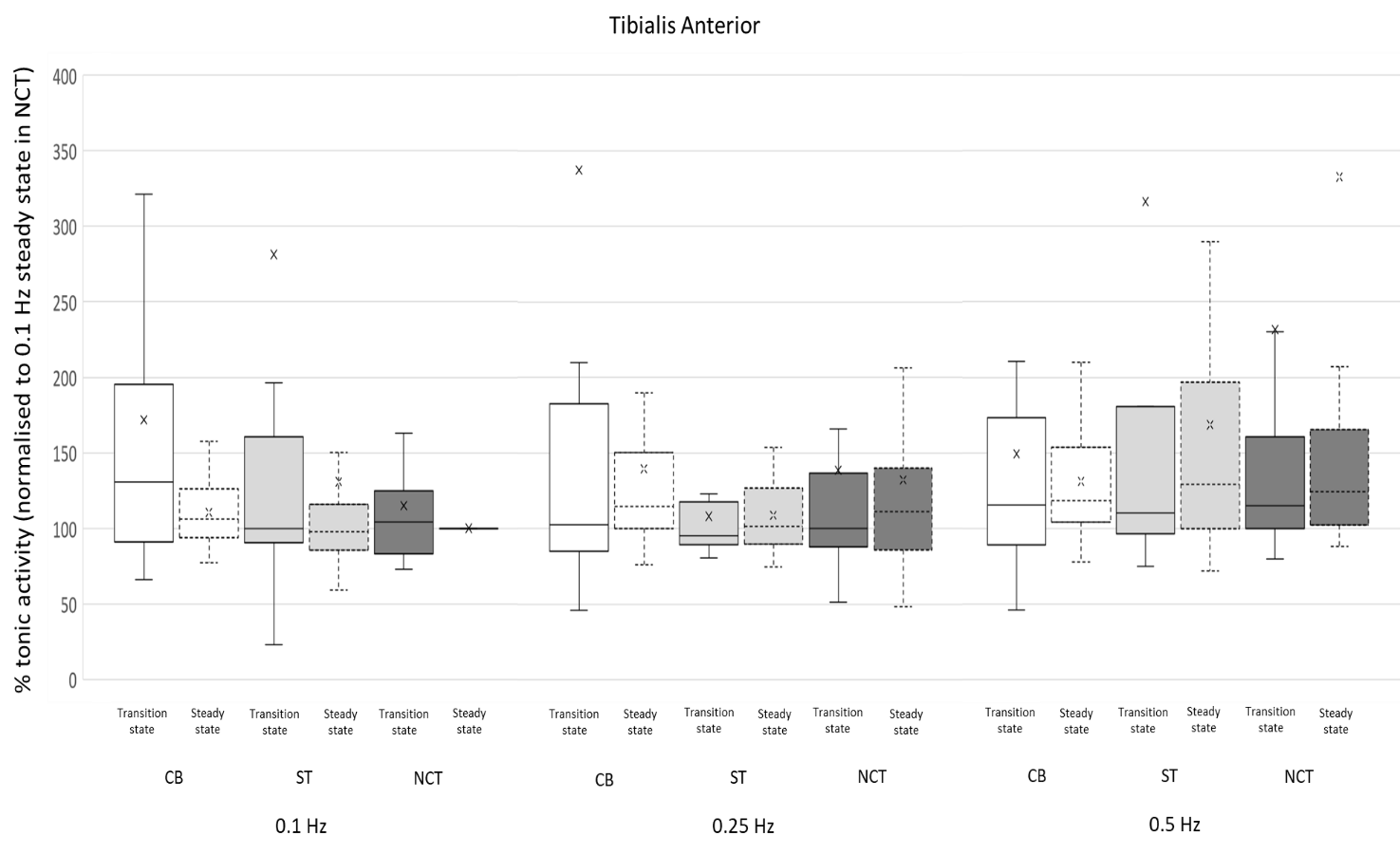
^[[1]](#footnote-1)^


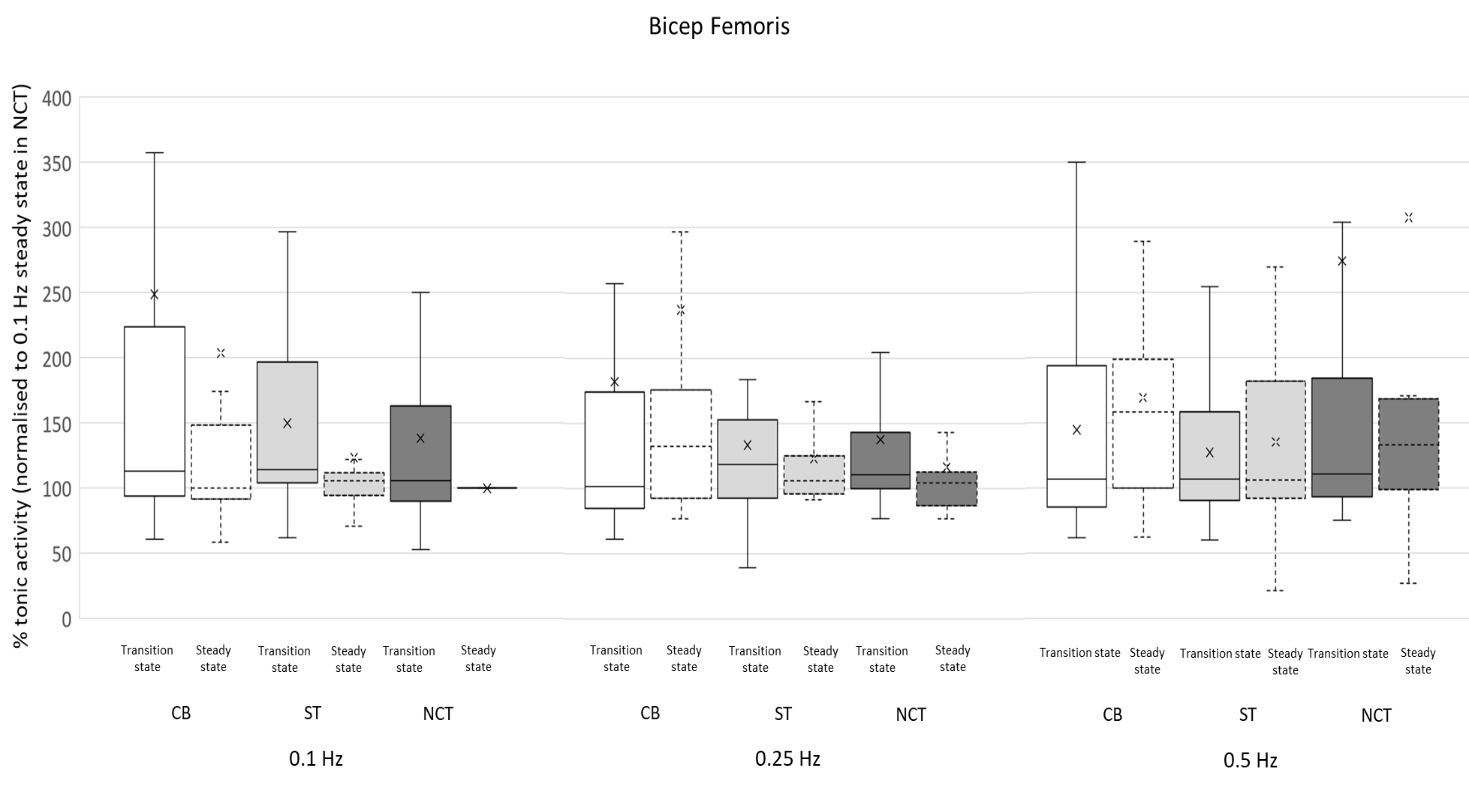
^[[2]](#footnote-2)^


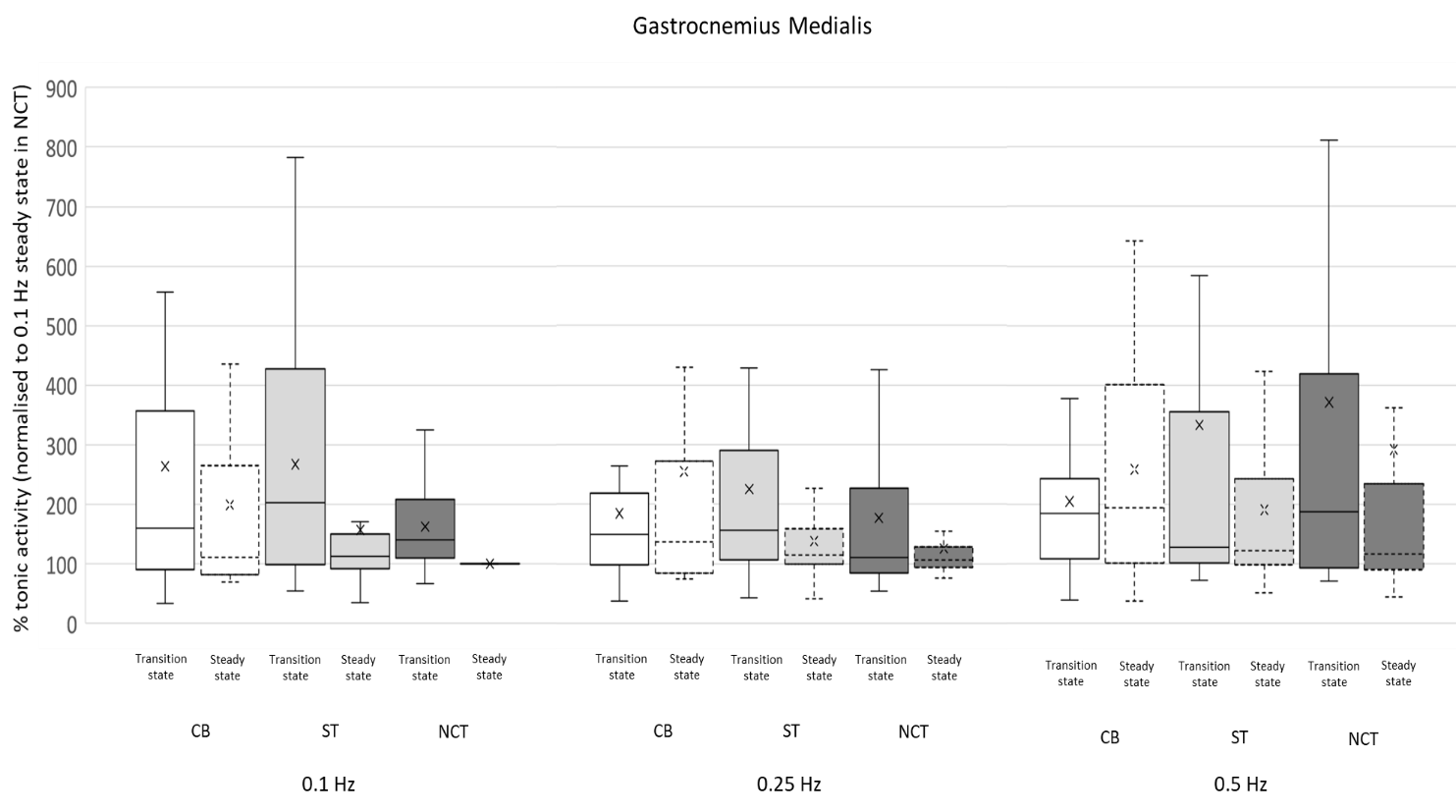
^[[3]](#footnote-3)^


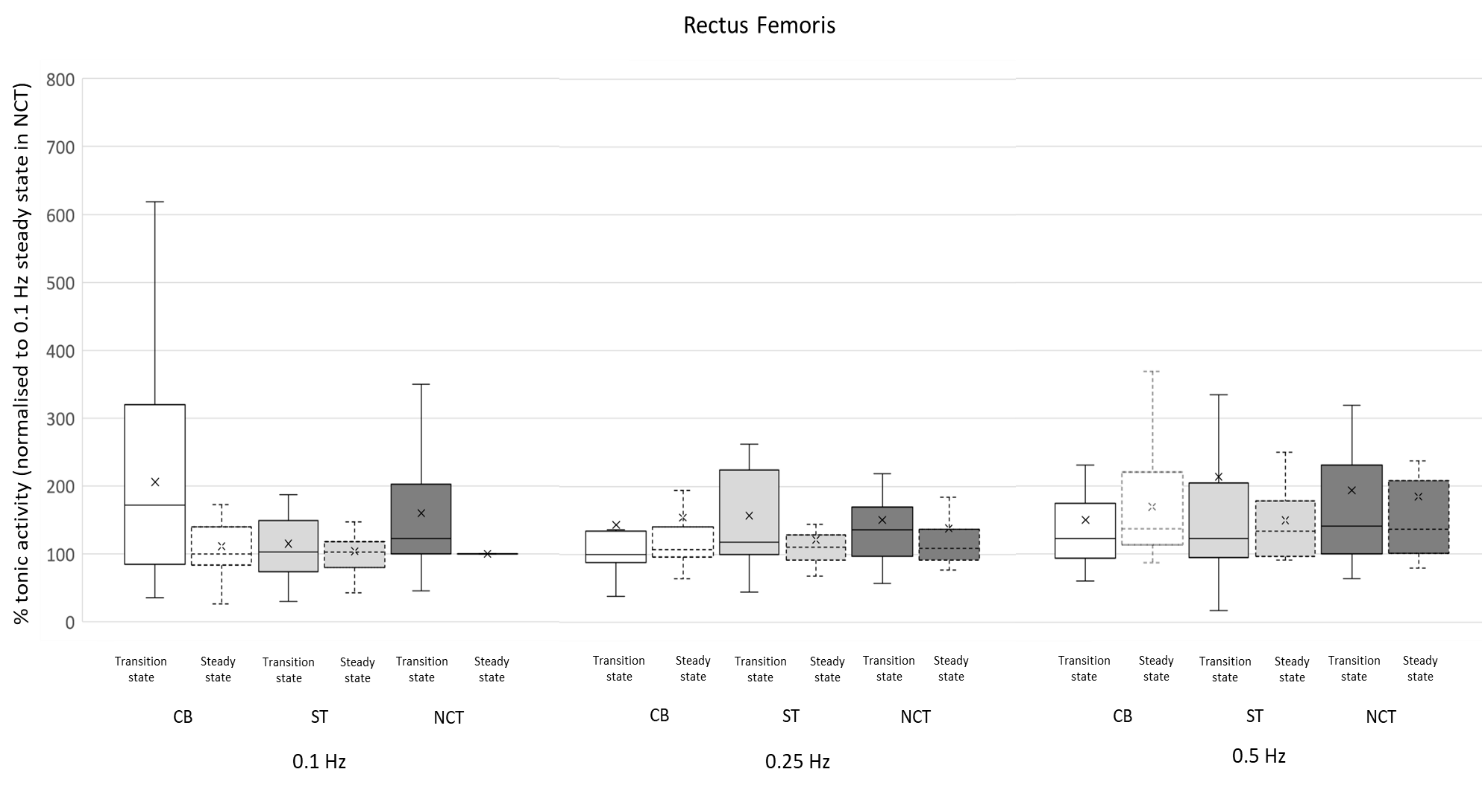
^[[4]](#footnote-4)^

1. Percent tonic activity (normalised to 0.1 Hz steady state in NCT) in transition and steady state for the Tibialis Anterior presented as box and whiskers with outliers removed. The x represents the mean, the horizontal line in the box represents the median, while the whiskers represent the minimum and maximum data point. [↑](#footnote-ref-1)
2. Percent tonic activity (normalised to 0.1 Hz steady state in NCT) in transition and steady state for the Bicep Femoris presented as box and whiskers with outliers removed. The x represents the mean, the horizontal line in the box represents the median, while the whiskers represent the minimum and maximum data point. [↑](#footnote-ref-2)
3. Percent tonic activity (normalised to 0.1 Hz steady state in NCT) in transition and steady state for the Gastrocnemius Medialis presented as box and whiskers with outliers removed. The x represents the mean, the horizontal line in the box represents the median, while the whiskers represent the minimum and maximum data point. [↑](#footnote-ref-3)
4. Percent tonic activity (normalised to 0.1 Hz steady state in NCT) in transition and steady state for the Rectus Femoris presented as box and whiskers with outliers removed. The x represents the mean, the horizontal line in the box represents the median, while the whiskers represent the minimum and maximum data point. [↑](#footnote-ref-4)
